# Editorial

**Published:** 1871-07

**Authors:** 


### Editorial.

***Original Communications.***

Our readers will notice with pleasure the increased value and extent of this department of the Journal. Indeed we have secured the assistance of a corps of co-laborers of which any journal might well be proud. Without invidious designation, we may safely say that, in the present number alone, there are individual articles worth more to the careful reader than a year's subscription to this or any other medical periodical in the country.

The variety of topics discussed is so great that we have not deemed it worth while to borrow matter from our contemporaries

We have several valuable communications on file, for which correspondents will accept thanks, and pardon us for temporary delay in appearance. Be not weary in well doing.

***The Prurient Prudes—***

long-haired men and short-haired women—who have taken it into their otherwise empty heads, to assume virtuous indignation at some of the expressions made use of by one of our correspondents, several months since, are confidentially advised to look over an article, published by one of our contemporaries in this city, entitled the "Social Evil." An attempt was made to have this latter article published at the municipal expense and sent to each family in the city.

Our correspondent's article was intended for physicians only, and we undertake to say, none of them were shocked. If any women read medical books or medical journals, we opine they have already lost the "wire edge" of their modesty sufficiently to endure even worse shocks.

A lady complimented Dr. Johnson, the lexicographer, on the fact that he had left out all the naughty words from his dictionary. "I perceive," said the great hater of shams, "that you have been looking for them."

Now you cream-colored man, or vinegar-visaged woman, who have taken it upon yourselves to howl over "VOLTA REEKE," do a farther service to the world by hanging yourselves respectively with a garter and a necktie.

***The Gaillard-Yandell***

imbroglio fills the papers of Louisville with unseemly professional squabbles. Meanwhile, we remain blissfully ignorant of the real facts in the case. Dr. Gaillard's promised *exposè* in the *Rich. and Lou. Jour.*, for June, has not yet come to hand. "In the name of the Prophet—Figs." The Presidency of the Am. Med. Association is getting to be a position fraught with as much danger from missiles of all nastiness as the old-time pillory. Let us have peace!

***Intestinal Concretion.***

J. P. Walker, M.D., of Mason City, Ill., favors us with a fragment of a fine specimen of a hair ball, encrusted with calculous matter, taken from the duodenum of a cow. The entire ball was eight and three-fourths inches in circumference. Such formations are not unprecedented, but are sufficiently rare to be considered curiosities.

***Radical Change.***

The Harvard Univ. Med. School has adopted a course of instruction extending, like the academic curriculum, throughout the year. Three years' instruction will be given by lectures, recitations, etc., and a series of examinations has been arranged, to occur at intervals during the course of study.

The plan is essentially the one first adopted and then abandoned by the University of Michigan, twenty odd years since. Practically it furnishes medical education to rich men's sons only. The vast majority of medical students have neither time nor means to avail themselves of the conceded advantages of such a course. Meanwhile, we reiterate, more depends on the teacher than the "plan." Not even Medical Students can gather figs from thistles.

***The State Medical Society.***

Proceedings at the recent session were promised us by the Secretary in season for the present number of the JOURNAL, but have not yet come to hand, and we are *reluctantly* obliged to go to press without them. Fortunately they will keep.

***Doctors in the United States.***

The total number of physicians who paid taxes to the government, for the year ending April 30, was 49,798. Of these there were regular or allopathic, 39,070; homœopathic, 2,961; hydropathic, 123; eclectic, 2,860; miscellaneous or not classified, 4,770.

